# Bandwidth-efficient and reliable communication in smart grid systems for modern energy networks using Trellis and Turbo Trellis Coded Modulation

**DOI:** 10.1038/s41598-026-54697-5

**Published:** 2026-06-08

**Authors:** Rna Ghallab, Mohamed Shaheen, Ahmed S. Elkorany, Somaya A. El-Feshawy

**Affiliations:** 1 Electrical Engineering Department, College of Engineering, Kafrelsheikh University, Kafr El-Sheikh 33516, Egypt; 2 Electronics and Electrical Comm. Eng. Dept., Faculty of Electronic Engineering, Menoufia University, Menouf 32952, Egypt; 3 Electronics and Electrical Comm. Eng. Dept. High Institute of Engineering, Belbis, Sharqia government, Egypt

**Keywords:** Smart grid power factor, TCM, TTCM, Spectral efficiency, Bit error detection and correction, Computational biology and bioinformatics, Engineering, Mathematics and computing

## Abstract

Smart Grids rely on robust communication infrastructures to monitor, control, and stabilize information in real time across geographically distributed energy resources. Trellis Coded Modulation (TCM) is a well-established technique for improving spectral efficiency and reliability, particularly in bandwidth-constrained and noisy channels. By combining convolutional coding with multilevel modulation, TCM achieves significant coding gains without increasing bandwidth, making it well suited for Smart Grid communication links. Turbo Trellis Coded Modulation (TTCM) extends TCM by incorporating parallel concatenated trellis encoders with iterative decoding, further enhancing performance and robustness under a wide range of channel distortions, including additive noise and fading. In this paper, we present the underlying mathematical framework for TCM and TTCM, simulation results under AWGN and Rayleigh fading channels, and comparisons to uncoded transmission. We also discuss future prospects of TCM, including integration with AI-driven adaptive coding and 5G-enabled Smart Grid infrastructures, highlighting the critical role of high-reliability communication systems in next-generation energy networks. Consequently, TTCM offers a hybrid solution that combines strong error correction with efficient bandwidth utilization, ensuring dependable communication for reliability-critical Smart Grid applications.

## Introduction

Modern Smart Grids rely on seamless communication among substations, IoT-enabled devices, and distributed renewable energy resources. Wireless communication channels in these environments are often challenged by multipath fading, impulsive noise, and cyber-physical security threats. Trellis Coded Modulation (TCM), which combines convolutional coding with signal set partitioning, improves error resilience while preserving bandwidth efficiency. Its ability to achieve coding gains without sacrificing spectral efficiency makes TCM particularly suitable for Smart Grid applications, such as phasor measurement unit (PMU) communications and demand response systems.

Turbo Trellis Coded Modulation (TTCM) extends TCM by incorporating parallel-concatenated encoders and iterative decoding, further enhancing reliability under noisy and fading channels. The iterative decoding mechanism allows TTCM to approach near-optimal error correction performance while maintaining bandwidth efficiency, making it well suited for low-latency, reliability-critical Smart Grid operations, including PMU data exchange and distributed energy resource (DER) coordination. This work presents the mathematical framework of TCM and TTCM, evaluates their performance through theoretical analysis and simulations, and compares them with conventional modulation and coding schemes to demonstrate their suitability for modern Smart Grid communication networks.

## Related work

Modern Smart Grids rely on advanced communication and control infrastructures to coordinate substations, distributed energy resources (DERs), and IoT-enabled devices. Wireless communication channels in these environments face challenges such as multipath fading, impulsive noise, and cyber-physical threats. Software-Defined Networking (SDN) enhances adaptability through intent-driven configurations, translating high-level objectives (e.g., load balancing, fault tolerance) into real-time network operations. This allows the grid to mitigate fluctuations, accommodate weather-dependent renewable generation, adapt resource allocation, and minimize outages^1–3^.

Machine Learning (ML) is transforming grid management, with Federated Learning (FL) enabling privacy-preserving model training across decentralized devices without sharing raw data. Techniques such as Low-Rank Adaptation (LoRA) reduce the computational and communication overhead of large-scale ML models, making them deployable on resource-constrained grid devices^2–4^. To address threats like malicious injection attacks, Generative Adversarial Networks (GANs) simulate attack scenarios for anomaly detection, while quantum-resistant cryptography ensures data integrity against advanced cyberattacks^5–7^.

Decentralized energy paradigms are gaining momentum, allowing consumers with distributed generation, including rooftop photovoltaic systems, to autonomously exchange excess electricity using blockchain-secured smart contracts. This reduces dependence on centralized utilities, increases cost savings, and enhances grid flexibility^8–10^. Vehicle-to-Everything (V2X) technologies, such as vehicle-to-grid (V2G) and vehicle-to-home (V2H), leverage electric vehicle (EV) batteries for mobile storage, enabling bidirectional energy flow that stabilizes the grid during peak demand and facilitates renewable integration^9–11^.

The deployment of 5G networks enhances grid communication with low latency (< 1 ms) and high bandwidth (up to 10 Gbps), enabling real-time applications such as phasor measurement units (PMUs) and IEC 61,850-based substation automation, which rely on precise synchronization for anomaly detection^12–14^. AI-driven control systems optimize renewable integration by suppressing voltage harmonics from inverters and wind generators, maintaining power quality^13–15^. Energy forecasting is improved through hybrid architectures combining Multi-access Edge Computing (MEC) and 5G, enabling real-time local predictions to inform dynamic pricing and demand response strategies^16–18^. Blockchain technology enhances transparency in peer-to-peer energy markets, ensuring secure and auditable transactions^19–22^. Data-driven load forecasting algorithms improve demand response effectiveness by encouraging consumers to shift usage to off-peak hours, reducing peak loads and avoiding costly infrastructure upgrades^23,20,21^.

Self-healing grids leverage deep learning models to detect faults from sensor networks and satellite imagery, triggering automated rerouting to isolate outages and restore service within minutes^22,24,25^. Smart meters provide granular consumption data for cloud-based analytics, which, when integrated with digital twins of physical grid assets, enable proactive load management and scenario simulation, such as EV charging surges or solar generation shortfalls^26–28^. Decentralized microgrids improve resilience by operating autonomously during grid outages, typically powered by hybrid renewable-storage systems optimized through Model Predictive Control (MPC) and Multi-Agent Systems (MAS)^27–29^. Advances in battery technologies, including high-energy lithium-ion and flow batteries, focus on improving energy density and cycle life to mitigate intermittency and stabilize frequency and voltage^30–32^.

The integration of variable renewable energy sources (VRES) continues to be a key research focus. AI-based forecasting improves the accuracy of solar and wind generation predictions, facilitating optimal dispatch planning^13–17^. Smart inverters provide reactive power support and voltage regulation to maintain power quality as VRES penetration increases^33–35^. Emerging storage technologies, including solid-state batteries, vanadium flow cells, and hybrid systems, support long-term reliability by smoothing intermittency and stabilizing grid frequency and voltage^5–9^. Social and economic considerations, such as customer engagement, demand response incentives, and dynamic pricing, further support the development of prosumer-driven decentralized energy markets^36,37^. Regulatory alignment remains essential to standardize protocols, ensure interoperability, and incentivize modernization^38–41^.

Grid optimization relies on advanced computational methods such as evolutionary algorithms and mixed-integer linear programming (MILP) for voltage control, topology reconfiguration, and operational cost reduction. Edge computing reduces latency by processing data locally at substations, critical for real-time operations. IoT sensors provide continuous monitoring of grid variables (e.g., voltage, frequency), feeding predictive maintenance and fault detection systems^42–44^. AI models, including Convolutional Neural Networks (CNNs) and Long Short-Term Memory (LSTM) networks, analyze these data streams to predict transformer failures or insulator degradation, minimizing maintenance costs. Cyber-physical systems combine grid hardware with digital control, including intrusion detection systems (IDS) that leverage machine learning to detect unauthorized activity and isolate compromised nodes during cyber-attacks^43–45^.

Hybrid energy storage systems, combining lithium-ion batteries and supercapacitors, are explored for frequency regulation and rapid discharge, while hydrogen storage supports long-term energy balancing. These integrated solutions maintain reliability, support grid stability, and enhance resilience in the face of growing renewable penetration and evolving cyber-physical threats.

## System architecture and smart grid communication model

This paper adopts a Smart Grid system model representing an integrated power system, where the electrical infrastructure is enhanced through monitoring, communication, and control functions to support reliable and efficient operation under increasing system complexity. The model emphasizes power system operation, network behavior, and system-level coordination, consistent with the scope of modern energy systems and electrical engineering.

**Fig. 1:**
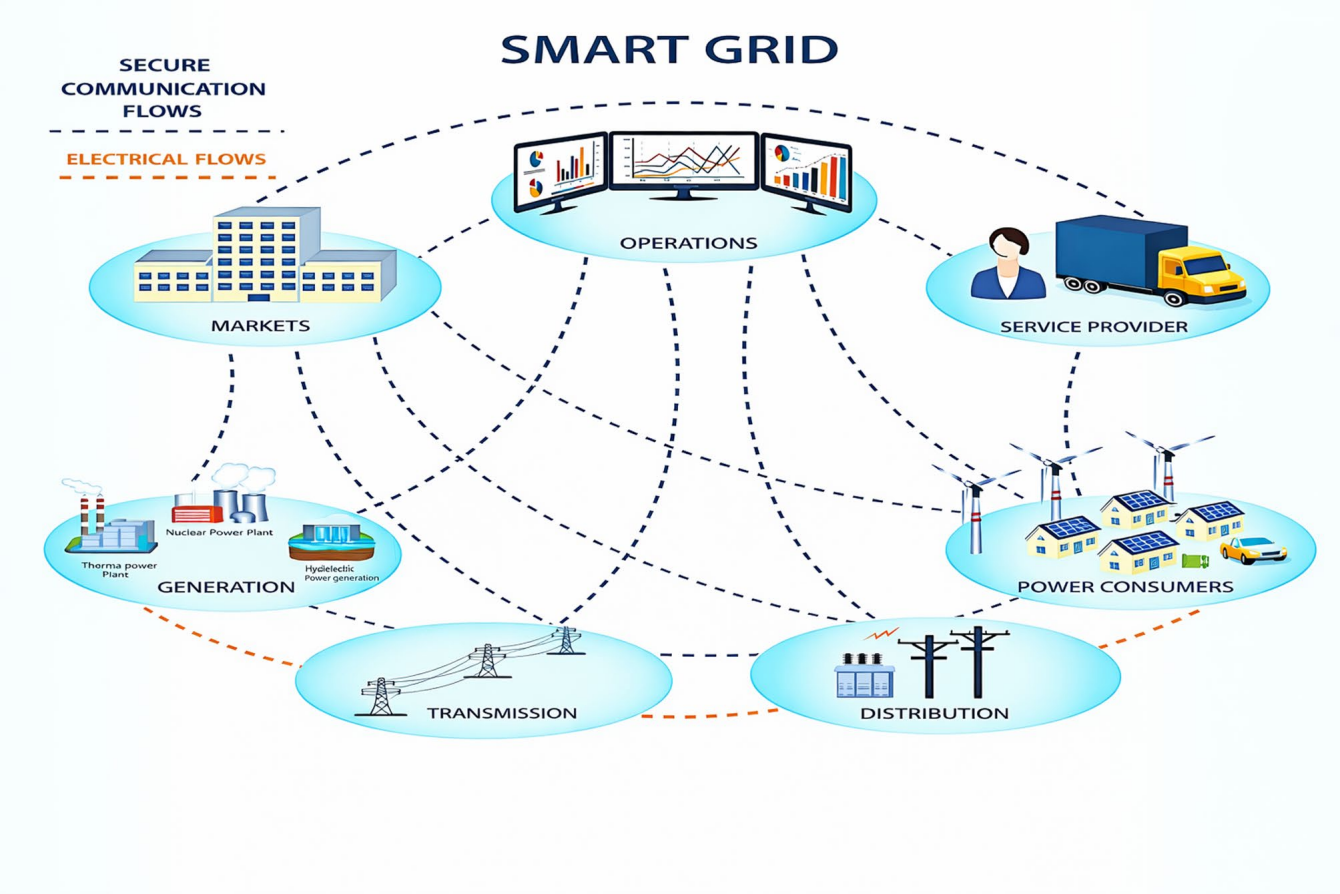
Smart grid system model.

As shown in Fig. 1, the system is structured into interacting domains, including generation, transmission, distribution, electricity consumers, operational control, markets, and service providers. These domains are interconnected through the physical electrical network and an overlaid communication layer, which enables timely information exchange necessary for system monitoring and coordinated control.

Reliable and bandwidth-efficient communication is foundational for modern Smart Grid infrastructures, where heterogeneous devices—such as substations, smart meters, phasor measurement units (PMUs), and distributed energy resources (DERs)—must exchange time-critical information under adverse channel conditions. Recent research emphasizes the integration of advanced communication techniques with grid-aware system design to meet the stringent latency, reliability, and scalability requirements of cyber-physical energy networks.

The evolution of Smart Grids has been strongly influenced by software-defined networking (SDN), intent-based networking, and edge computing architectures, enabling adaptive control, fault tolerance, and dynamic resource allocation across large-scale power networks. Machine learning (ML) and federated learning (FL) approaches further enhance grid intelligence by enabling decentralized data processing while preserving consumer privacy. Simultaneously, the proliferation of renewable energy sources, electric vehicles, and peer-to-peer energy trading has increased communication traffic and exposed networks to fading, interference, and cyber-physical threats.

From a physical-layer perspective, ensuring robust communication over bandwidth-limited and fading-prone channels is a critical challenge. Conventional modulation schemes without channel coding provide limited resilience in such environments, motivating the adoption of bandwidth-efficient coded modulation techniques. Trellis Coded Modulation (TCM), introduced by Ungerboeck, combines convolutional coding with multilevel modulation to achieve significant coding gains without bandwidth expansion, making it highly suitable for Smart Grid communication channels.

### Electricity system structure

The electrical infrastructure follows a hierarchical structure comprising generation, transmission, and distribution networks, with increasingly decentralized and bidirectional power flows. Energy is produced by centralized conventional plants and distributed renewable generation units. Large-scale generation is typically connected to the transmission network, while DERs such as rooftop solar and small generators are integrated primarily at the distribution level. Transmission networks enable bulk power delivery and inter-regional exchange while maintaining stability and reliability. Distribution systems are transitioning from passive operation to active network management due to the integration of DERs and flexible loads.

### Communication and measurement infrastructure

A communication and measurement infrastructure overlays the electrical network to provide system visibility and enable coordinated operation. This infrastructure supports acquisition and exchange of measurements, status information, and control signals between generating units, network components, and control centers. Timely and reliable communication is essential to maintain voltage and frequency within acceptable limits, manage congestion, and respond to disturbances. Communication capabilities thus serve as an enabling layer for advanced monitoring and control rather than as an independent objective.

### System operation and control

System operations are coordinated through operational domains, including control centers and management platforms such as Supervisory Control and Data Acquisition (SCADA), Energy Management Systems (EMS), and Distribution Management Systems (DMS). These domains are responsible for real-time monitoring, state estimation, contingency analysis, and operational decision-making. Operational control actions rely on measurements from the physical system and are executed according to safety and reliability criteria. Coordination between transmission and distribution operations is especially important in the presence of high DER penetration.

### Load, distributed resources, and demand response

The consumer sector includes residential, commercial, and industrial loads, as well as prosumers equipped with local generation and storage. Advanced metering and local control systems allow loads to adjust consumption according to system conditions or economic signals. Flexible loads and DERs are considered controllable resources that, when coordinated by system operators, contribute to load balancing, voltage support, and overload management.

### Market links and support services

Market mechanisms provide an economic framework to coordinate generation and consumption while supporting system operation. These include energy trading and ancillary services, where participants submit bids and offers subject to technical constraints. Market outcomes, including dispatch plans and price signals, inform operational decisions and resource utilization. Market interactions are considered together with physical network constraints to ensure safe and reliable system operation.

### Service providers

Service providers deliver specialized functions such as metering, maintenance, and asset management. While reliant on system measurements and operational data, these services are subordinate to core operational and control objectives.

### Integrated power system perspective

The interaction of power infrastructure, measurement systems, and control mechanisms enables the Smart Grid to be viewed as an integrated power system with enhanced observability and controllability. This integrated perspective facilitates higher penetration of renewable energy sources, distributed generation, and flexible demand management, addressing central challenges in modern power systems research.

## Mathematical model

This section presents a comprehensive mathematical framework for analyzing wireless communication links in smart grid environments. The model incorporates large-scale path loss, small-scale fading, co-channel interference, and additive noise, enabling the evaluation of received signal power, signal-to-interference-plus-noise ratio (SINR), and channel capacity under realistic deployment conditions.

### Signal model

Let x(t) be the transmitted signal and y(t) the received signal. The baseband equivalent received signal can be expressed as:

1 $${\mathrm{y}}\left( {\mathrm{t}} \right)\,=\,{\mathrm{h}}\left( {\mathrm{t}} \right){\mathrm{x}}\left( {\mathrm{t}} \right)\,+\,{\mathrm{n}}\left( {\mathrm{t}} \right))$$

where h(t) is the channel gain, accounting for path loss and fading, and n(t) is complex additive white Gaussian noise (AWGN) with variance σ_n_. This model assumes flat, slow fading, which is typical for low-mobility smart grid nodes. Carrier frequency offsets are considered negligible under this scenario.

### Path loss and link budget

The received signal power depends on path loss, which is modeled empirically as:

2 $${\mathrm{PL}}\left( {\mathrm{d}} \right)={\mathrm{P}}{{\mathrm{L}}_0}+{\mathrm{1}}0{\text{n lo}}{{\mathrm{g}}_{{\mathrm{1}}0}}\left( {{\mathrm{d}}/{{\mathrm{d}}_{\mathrm{o}}}} \right)\,+\,{\mathrm{Xss}}$$

where PL(d) is the total path loss at distance d in dB, PL_0_ is the reference path loss at d_0_ = 10 m, n is the path loss exponent, and Xss represents small-scale fading, which may follow Rayleigh or Nakagami-m distributions.

The received signal power is obtained from the link budget as expressed :

3 $${\mathrm{Pr}}\left( {\mathrm{d}} \right){\text{ }}\left[ {{\mathrm{d}}{{\mathrm{B}}_{\mathrm{m}}}} \right]\,=\,{\text{Pt }}\left[ {{\mathrm{d}}{{\mathrm{B}}_{\mathrm{m}}}} \right]\,+\,{\mathrm{Gt}}+{\mathrm{Gr}}\, - \,{\mathrm{PL}}\left( {\mathrm{d}} \right)$$

where P_t_ is the transmit power and G_t_ and G_r_ are the transmitter and receiver antenna gains, respectively. This formulation ensures accurate received power estimation, especially for short-range smart grid communication.

### Fading Model: Nakagami-m

Small-scale fading Xss is modeled using a Nakagami-m distribution, which generalizes Rayleigh fading:

4 $${{\mathrm{f}}_{\mathrm{r}}}\left( {\mathrm{r}} \right){\text{ }}={\text{ }}[{\mathrm{2}}{{\mathrm{m}}^{\mathrm{m}}}/\Gamma \left( {\mathrm{m}} \right){\Omega ^{\mathrm{m}}}]{\text{ }}{{\mathrm{r}}^{{\mathrm{2m-1}}}}{\text{exp }}( - \,{\mathrm{m}}{{\mathrm{r}}^2}/\Omega ),{\text{ where r}}\, \geqslant \,0{\text{ }}$$

where m ≥ 1/2 is the Nakagami fading parameter, Ω = E[r^2^] denotes the average received signal power, and Γ(.) represents the Gamma function.

The parameter m controls the severity of fading. Specifically, m = 1 corresponds to Rayleigh fading, which is suitable for modeling non-line-of-sight (NLOS) smart grid environments, while m > 1 represents less severe fading conditions typically associated with line-of-sight (LOS) links, such as substation-to-control-center communications. Conversely, m < 1 characterizes more severe fading scenarios encountered in dense urban or indoor smart metering deployments.

### SINR analysis

Considering interference I from neighboring nodes and noise N, the instantaneous SINR is given by:

5 $${\mathrm{SINR}}=[{\mathrm{Pr}}\mid {\mathrm{h}}{\mid ^{\mathrm{2}}}\left] / \right[{\text{ I}}\,+\,{\text{N }}]$$

For Nakagami-mmm fading, the PDF of the SINR can be expressed as:

6 $${\text{F SINR}}(\gamma )=[{{\mathrm{m}}^{\mathrm{m}}}/\Gamma \left( {\mathrm{m}} \right){\gamma ^{{ {-m}}}}]{\gamma ^{{\mathrm{m}} - {\mathrm{1}}}}{\mathrm{exp}}( - \,{{ {m}}_\gamma }/{\gamma ^ - }),\gamma \, \geqslant \,0$$

where $\gamma ^{ - }$ = P_r_/(I + N) is the average SNR. This formulation enables evaluation of link reliability, outage probability, and system-level performance metrics.

### Channel capacity

The channel capacity under AWGN and fading is given by Shannon’s formula:

7 $$C={\mathrm{Blo}}{{\mathrm{g}}_{\mathrm{2}}}({\mathrm{1}}\,+\,{\mathrm{SINR}})$$

where B is the allocated bandwidth. Spectral efficiency (SE) is defined as ) SE=log_2_(1 + SINR) in bits/s/Hz. This allows evaluation of achievable data rates for narrowband, wideband, and broadband smart grid channels.

### Assumptions summary

Carrier frequency offset (CFO) is neglected in this work due to the low-mobility and quasi-static nature of Smart Grid communication links. Smart Grid nodes are either stationary or exhibit extremely limited mobility, resulting in negligible Doppler shifts compared to conventional mobile communication systems. In addition, Smart Grid communication typically operates over narrowband channels with stable oscillators and network-level synchronization, which further suppresses residual frequency offsets. Under these conditions, the channel coherence time is significantly larger than the symbol duration, allowing any minor frequency offsets to be effectively compensated or safely ignored without impacting the validity of the proposed analysis.

The channel is assumed to exhibit flat, slow fading, typical of low-mobility nodes. Noise is modeled as AWGN with variance σ_n_^2^. Aggregate interference from neighboring nodes is included. Path loss follows an empirical model calibrated to environmental observations. Carrier frequency offsets are negligible, and fading follows Nakagami-m, with Rayleigh fading as a special case when m = 1.

Based on the system and channel model presented in section “System architecture and smart grid communication model”, the following sections investigate coding techniques capable of enhancing reliability and spectral efficiency under smart grid constraints.”

## Trellis Coded Modulation (TCM)

**Fig. 2:**
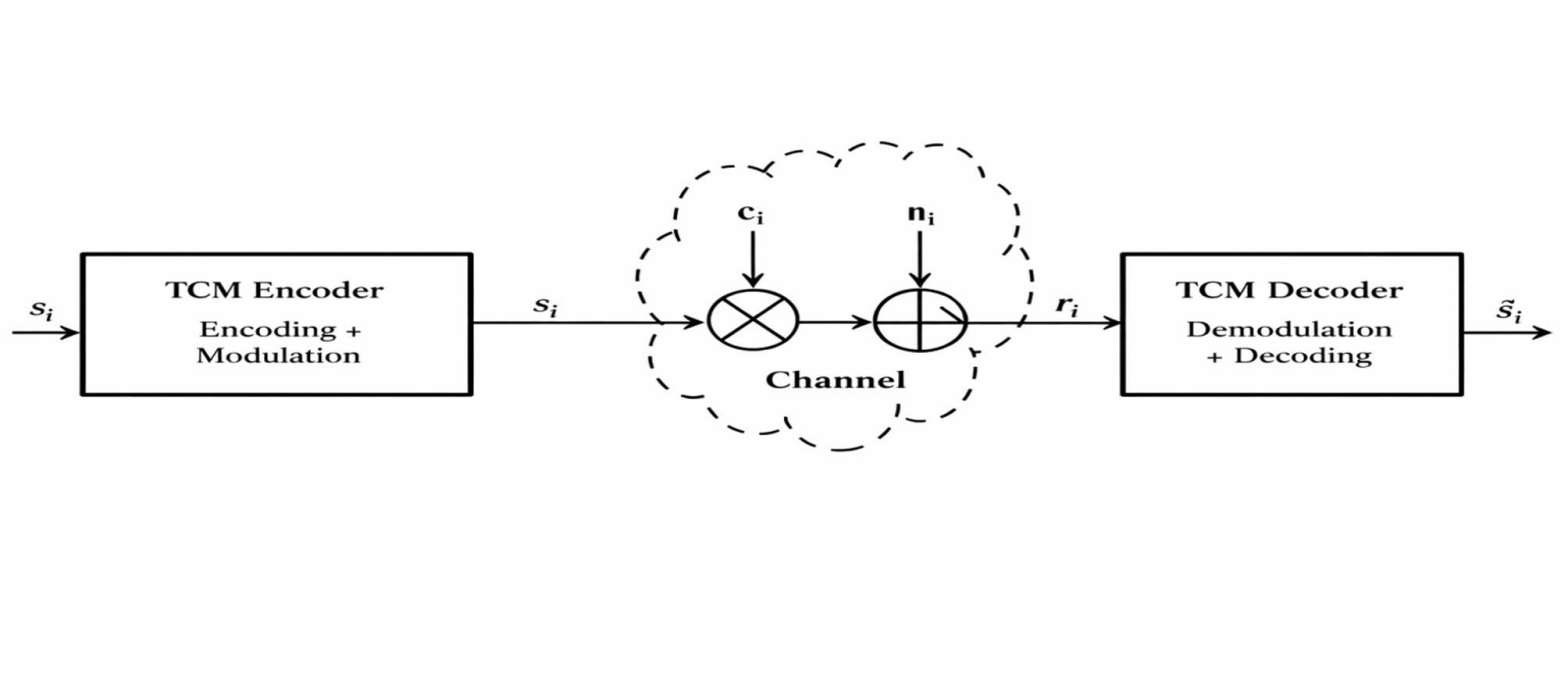
TCM block diagram.

TCM Trellis-Coded Modulation primarily maximizes spectral efficiency by combining convolutional coding with multilevel modulation, achieving significant coding gains without expanding bandwidth. However, conventional modulation schemes without channel coding are limited in harsh communication environments, motivating the use of bandwidth-efficient coded modulation techniques. Turbo Trellis-Coded Modulation (TTCM) extends TCM by employing parallel-concatenated trellis encoders and iterative decoding, offering enhanced error resilience under severe fading and low-SNR conditions. While TCM provides higher spectral efficiency, TTCM is evaluated for its robustness and reliability, making it particularly suited for reliability-critical Smart Grid communication links where consistent performance and reduced packet loss are prioritized over peak-throughput optimization. As illustrated in Fig. 2, TCM schemes such as BPSK, QPSK, and QAM are utilized to improve performance in bandwidth-limited channels, while TTCM highlights the trade-off between complexity and operational reliability.

In the transmitter, the input bit stream is first processed by a Trellis-Coded Modulation (TCM) encoder, which combines convolutional coding with modulation (e.g., BPSK, QPSK, or QAM) to add redundancy and improve error resilience without expanding bandwidth. The encoded symbols are then transmitted over a wireless channel, where they are subject to fading, path loss, and additive noise.

At the receiver, the corrupted signal is first demodulated to recover the transmitted symbols and then passed through a TCM decoder, which uses the trellis structure to correct errors introduced by the channel. In the case of Turbo Trellis-Coded Modulation (TTCM), parallel-concatenated trellis decoders iteratively exchange extrinsic information to further enhance reliability, producing an estimated output bit stream that closely matches the original input, thereby ensuring robust and reliable communication under adverse Smart Grid channel conditions.

### Mathematical model

#### Channel Gain (CG)

Channel Gain (CG), which is defined as the ratio of the required energy for uncoded transmission to that for coded transmission at the same error rate, given by:

8 $${\mathrm{Gain}}=\left\{ {\left( {\frac{{{{\mathrm{E}}_{{\mathrm{Uncoded}}}}}}{{{{\mathrm{E}}_{{\mathrm{Coded}}}}}}} \right).\left( {\frac{{{\mathrm{d}}_{{{\mathrm{f~~Uncoded}}}}^{2}}}{{{\mathrm{d}}_{{{\mathrm{f~~Codedd}}}}^{2}}}} \right)} \right\}~$$

Where

${\mathrm{~}}{{\mathrm{E}}_{{\mathrm{Coded}},{\mathrm{Uncoded}}}}$. Normalized average received energy for uncoded and coded. ${\mathrm{d}}_{{{\mathrm{f~Coded}},{\mathrm{Uncoded}}}}^{2}$ Minimum squared free distance of the code.

The channel gain quantifies the SNR improvement provided by the coded over the uncoded for the same error probability.

#### BER approximation in AWGN

9 $${\mathrm{Bit~Error~Rate}} \approx {\mathrm{Q~}}\left( {\sqrt {\frac{{{\mathrm{d}}_{{\mathrm{f}}}^{2}{{\mathrm{E}}_{\mathrm{s}}}}}{{2{{\mathrm{N}}_0}}}} } \right){\mathrm{~}}$$

Where:

$d_{f}^{2}$: Minimum squared free distance of the code. ${E_s}$: Symbol energy. ${N_0}$: One-sided noise power spectral density. Q: Gaussian Q-function, probability of normal distribution.

## Turbo Trellis Coded Modulation (TTCM)

Turbo Trellis Coded Modulation (TTCM) extends the conventional Trellis Coded Modulation (TCM) framework by employing parallel-concatenated trellis encoders and iterative decoding based on the turbo principle. While TTCM has been widely studied in broadband and cellular communication systems, its application to Smart Grid environments remains relatively underexplored. In Smart Grid communication scenarios—characterized by long operational lifetimes, quasi-static channels, and stringent reliability requirements—the stability and robustness of iterative decoding are often more critical than achieving peak spectral efficiency. Unlike conventional broadband networks, Smart Grid links prioritize predictable performance, long-term reliability, and resilience to fading over high throughput. TTCM is therefore particularly suitable for Smart Grid deployments, as its strong error-correction capability under low-SNR and fading-limited conditions reduces packet loss for critical monitoring and control messages, directly enhancing overall system reliability.

**Fig. 3:**
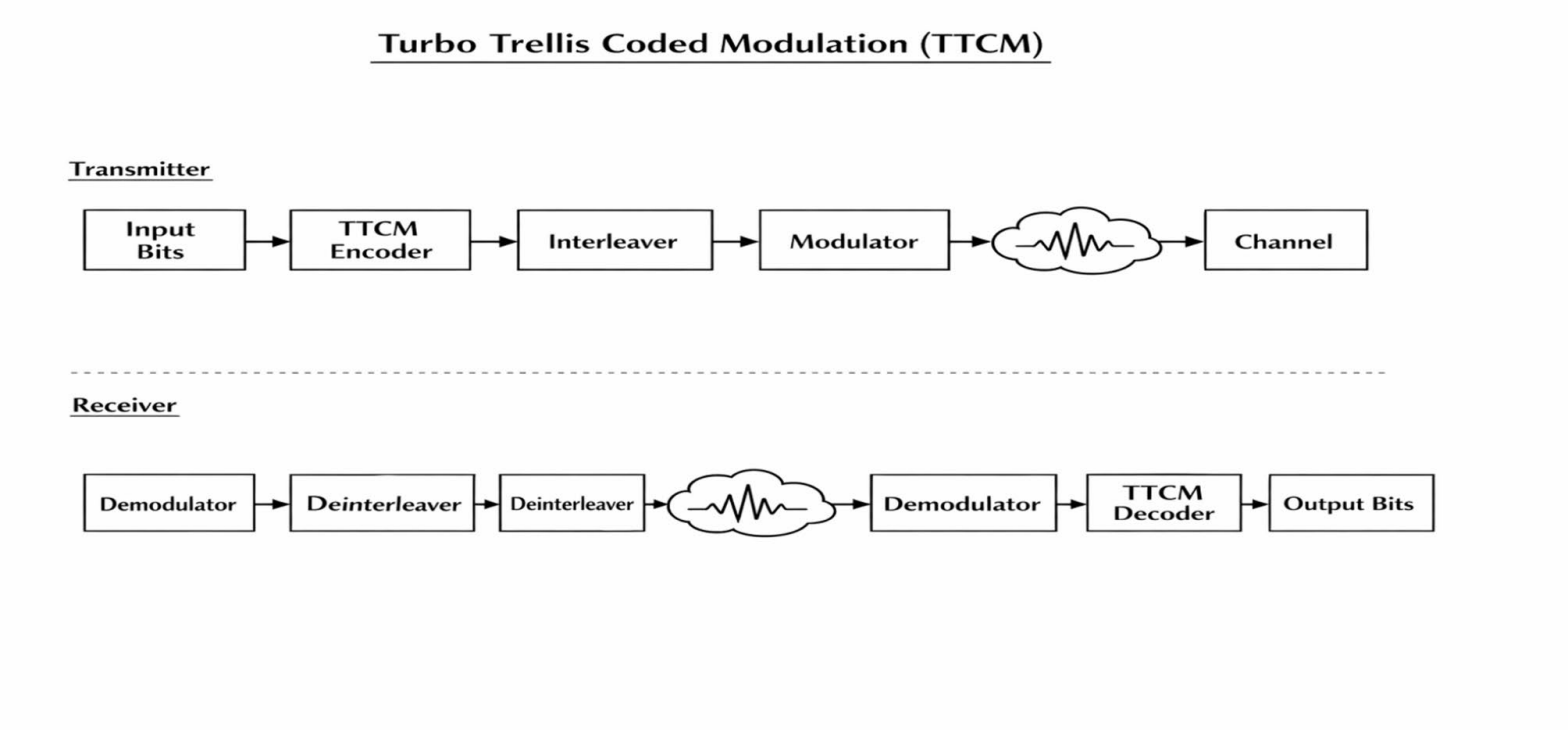
TTCM block diagram.

As illustrated in Fig. 3, TTCM combines the advantages of turbo coding and TCM to achieve high throughput with reduced error probability while maintaining efficient bandwidth utilization. This joint coding–modulation approach provides strong error correction capability without requiring additional bandwidth, making it particularly suitable for reliability-critical communication systems.

**Transmitter side**:

At the transmitter, the input bit stream is processed by a Turbo Trellis Coded Modulation encoder composed of two parallel Recursive Systematic Convolutional (RSC) encoders separated by an interleaver. The interleaver permutes the input bits to reduce correlation between the encoder outputs and enhance error correction performance. The encoded bit streams are subsequently mapped onto modulation symbols (e.g., QPSK or QAM) according to the selected modulation scheme and transmitted over the wireless channel, which may introduce noise, fading, and other impairments. This architecture enables the simultaneous exploitation of coding gain and modulation efficiency, thereby improving link reliability without sacrificing spectral efficiency.

**Receiver side**:

At the receiver, the incoming signal is first processed by a soft-input demodulator that generates probabilistic metrics, such as log-likelihood ratios (LLRs), for the transmitted bits. These soft values are fed into the iterative TTCM decoder, where the two constituent decoders exchange extrinsic information through multiple decoding iterations. Interleaving and deinterleaving operations ensure proper alignment of information between the decoders. Upon convergence, the decoder produces an estimate of the transmitted bit stream with significantly reduced error probability. This iterative decoding process leverages the turbo principle to approach near-optimal performance under noisy and fading channel conditions, which is particularly beneficial for reliability-critical Smart Grid monitoring, control, and protection applications.

### Mathematical model

#### Bit Error Rate (BER) approximation

10 $${\mathrm{BER}} \approx {\mathrm{T}}\left( {\mathrm{Y}} \right){\mid _{{\mathrm{Y}}={{\mathrm{e}}^{ - \left( {\frac{{{{\mathrm{E}}_{\mathrm{b}}}}}{{4{{\mathrm{N}}_0}}}} \right)}}}}~$$

Where:

${\mathrm{T}}\left( {\mathrm{Y}} \right)$: Transfer function of the convolutional code. ${{\mathrm{E}}_{{\mathrm{b}}/{{\mathrm{N}}_0}}}$: Energy per Noise power.

## Simulation results

Quadrature Phase Shift Keying (QPSK) modulation is employed in conjunction with the proposed TTCM scheme due to its favorable trade-off between spectral efficiency and robustness under low-SNR and fading conditions typical of Smart Grid communication links. In addition, 16-QAM modulation is considered to evaluate system performance across different robustness–throughput trade-offs relevant to diverse Smart Grid application requirements. The performance of both TCM and TTCM schemes is evaluated in the context of Smart Grid communication devices using a grid-oriented link capacity formulation combined with a realistic propagation path-loss model as illustrated in table 1. Moderate MIMO configurations, specifically 2 × 2 and 4 × 4, are adopted to represent practical deployment scenarios such as substation-to-substation and aggregator-to-smart-meter links, where cost, hardware limitations, and synchronization constraints render massive MIMO impractical. While TCM offers higher spectral efficiency in bandwidth-limited scenarios, TTCM is incorporated to highlight the reliability–complexity trade-off, as its iterative decoding process yields improved BER performance and enhanced robustness under fading conditions, which is essential for reliability-critical Smart Grid monitoring, control, and protection signals. 

**Table 1:** The simulation model specifications.

Criterion	Features
Path loss model	As in Eqs. (2) and (3, 4)
Path loss exponent (η)	2.5 dB
Distance (d)	10 m
Transmitted power	20 dBm
Amplifier gain (A_p_)	1 dB
Received signal strength	As in Eq. 1
Bit Error Rate (BER)	10^–3^
Modulation order	BPSK, QPSK,16QAM
Diversity codes	Trellis /Turbo Trellis Coded Modulation

**Fig. 4:**
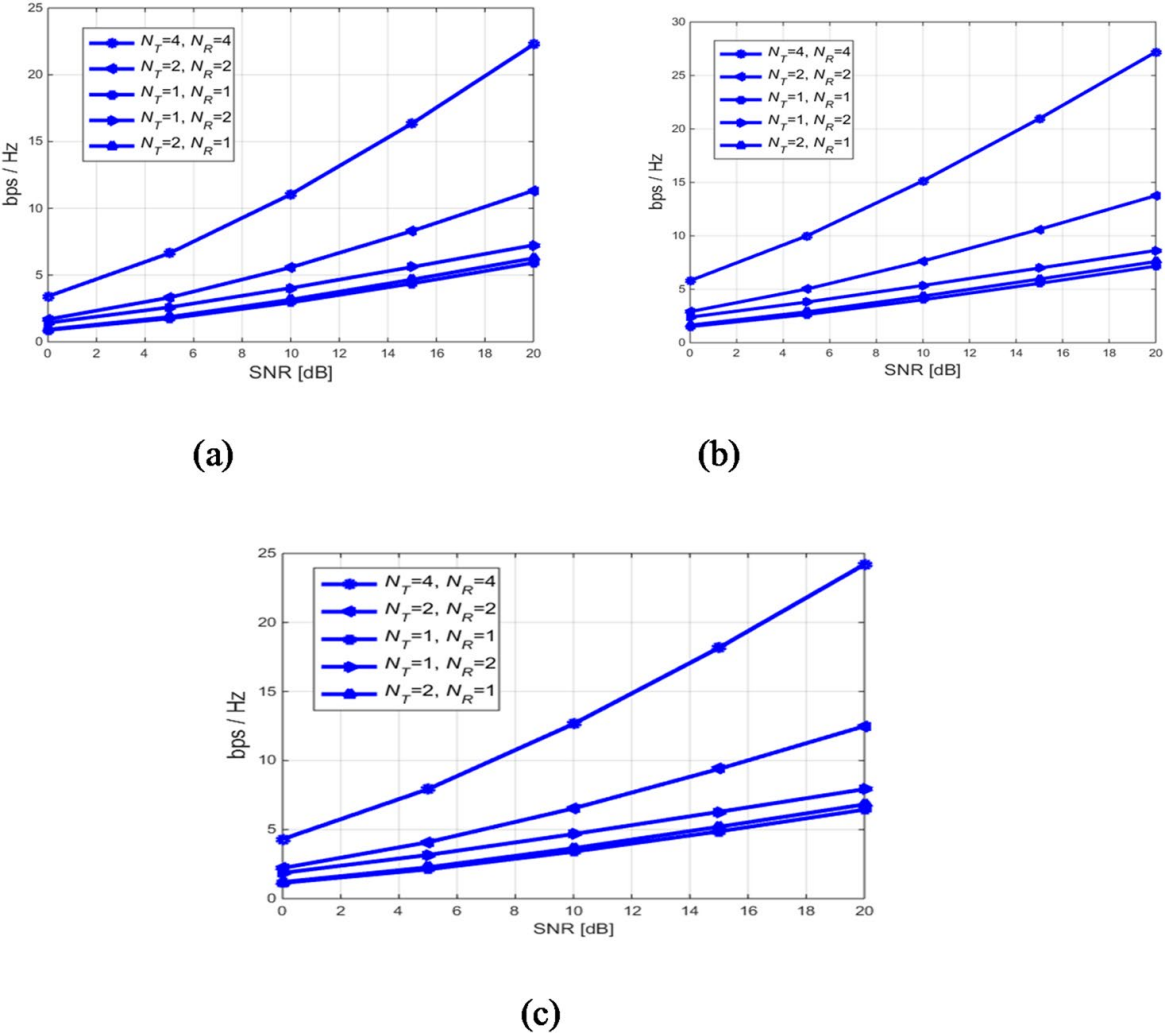
(a, b & c) where **(a)** spectral efficiency (bps/HZ) performance without coding technique under different SNR = 20 dB, (**b)** The spectral efficiency (bps/HZ) performance under different SNR = 20 dB, by using TCM Coding Method. (**c)** The spectral efficiency (bps/HZ) performance under different SNR = 20 dB, by using TTCM Coding method.

Figure 4 (a, b and c) and Table 2 compare the spectral efficiency performance at an SNR of 20 dB for uncoded, TCM coded and TTCM coded. As shown in Fig. 4(a), the unencoded system provides the lowest efficiency due to the lack of error correction. Furthermore, Fig. 4(b) shows that TCM coding improves performance by integrating modulation and coding to increase reliability without additional bandwidth. Finally, in Fig. 4(c), the TTCM scheme achieves the highest spectral efficiency, which benefits from iterative decoding and code diversity. Overall, TTCM provides the best results, while TCM provides moderate gains over the uncoded baseline, confirming the effectiveness of advanced coding techniques to improve throughput and robustness.

**Table 2:** For Fig. 4 (a, b, c): Spectral Efficiency at SNR = 20 dB.

System Type	SNR (dB)	Spectral Efficiency (bps/Hz)	Key Observation
(a) Uncoded	20	Baseline (Lowest) ~ 23 bps/Hz	No error-correction coding; serves as the reference performance
(b) TCM Coding		Highest ~27 bps/Hz	Superior to TCM and uncoded, leveraging turbo iterative decoding for near-optimal performance
(c) TTCM Coding		Improved ~ 24.8 bps/Hz	Higher efficiency than uncoded due to trellis coding gain

**Fig. 5:**
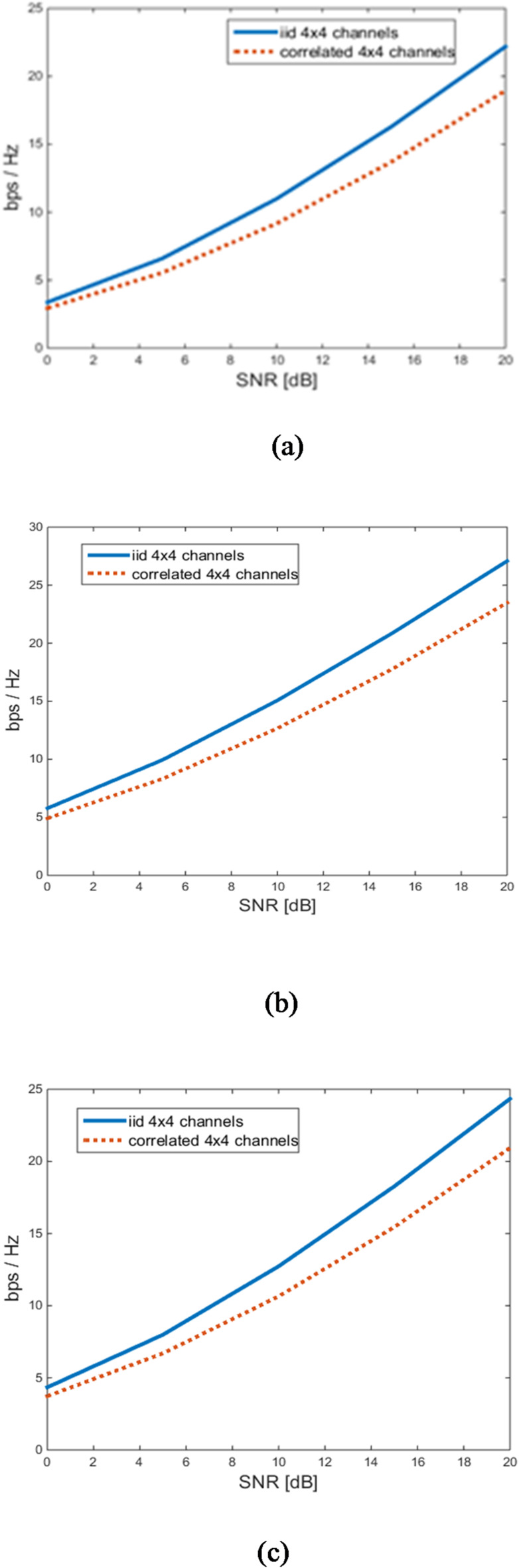
(a, b, c) where **(a)** Spectral efficiency opposed to SNR without coding technique at 4 × 4 channel, **(b)** Spectral efficiency against SNR with TCM coding method. **(c)** Spectral efficiency in opposition to SNR with various precoding methods by using TTCM Coding scheme.

Figure 5 (a, b, and c) and Table 3 compares the spectral efficiency versus SNR for uncoded, TCM-coded, and TTCM-coded with precoding over a 4 × 4 MIMO channel As shown in Fig. 5(a), the uncoded system provides the baseline performance (~ 12–22 bps/Hz), restricted by the absence of error-correction coding. In Fig. 5(b), the TCM-coded system achieves the highest spectral efficiency (~ 15–28 bps/Hz) due to its integrated modulation–coding design, which enhances error resilience without requiring additional bandwidth. Meanwhile, Fig. 5(c) shows the performance of the TTCM coded system with different precoding schemes, which provides moderate improvement (~ 13–24 bps/Hz); however, complexity and convergence factors slightly reduce its effectiveness compared to TCM. Overall, TCM illustrates the most efficient and robust performance for a 4 × 4 MIMO configuration, which are providing an optimal balance between throughputs, channel coding gain, and computational cost.

**Table 3:** For Fig. 5 (a, b, c): spectral efficiency vs. SNR (4 × 4 MIMO Channel).

Coding/Precoding	Channel	SNR (dB)	Spectral efficiency (bps/Hz)	Key observation
(a) Uncoded	4 × 4 MIMO	10–20	~ 12–22 bps/Hz (baseline)	Baseline; lowest performance across SNR range
(b) TCM Coding		10–20	~ 15–28 bps/Hz	Highest performance: TCM’s joint coding- modulation enhances error resilience
(c) TTCM + Precoding		10–20	~ 13–24 bps/Hz (varies by scheme)	Moderate gains over uncoded, but underperforms vs. TCM; complexity trade-offs

**Fig. 6:**
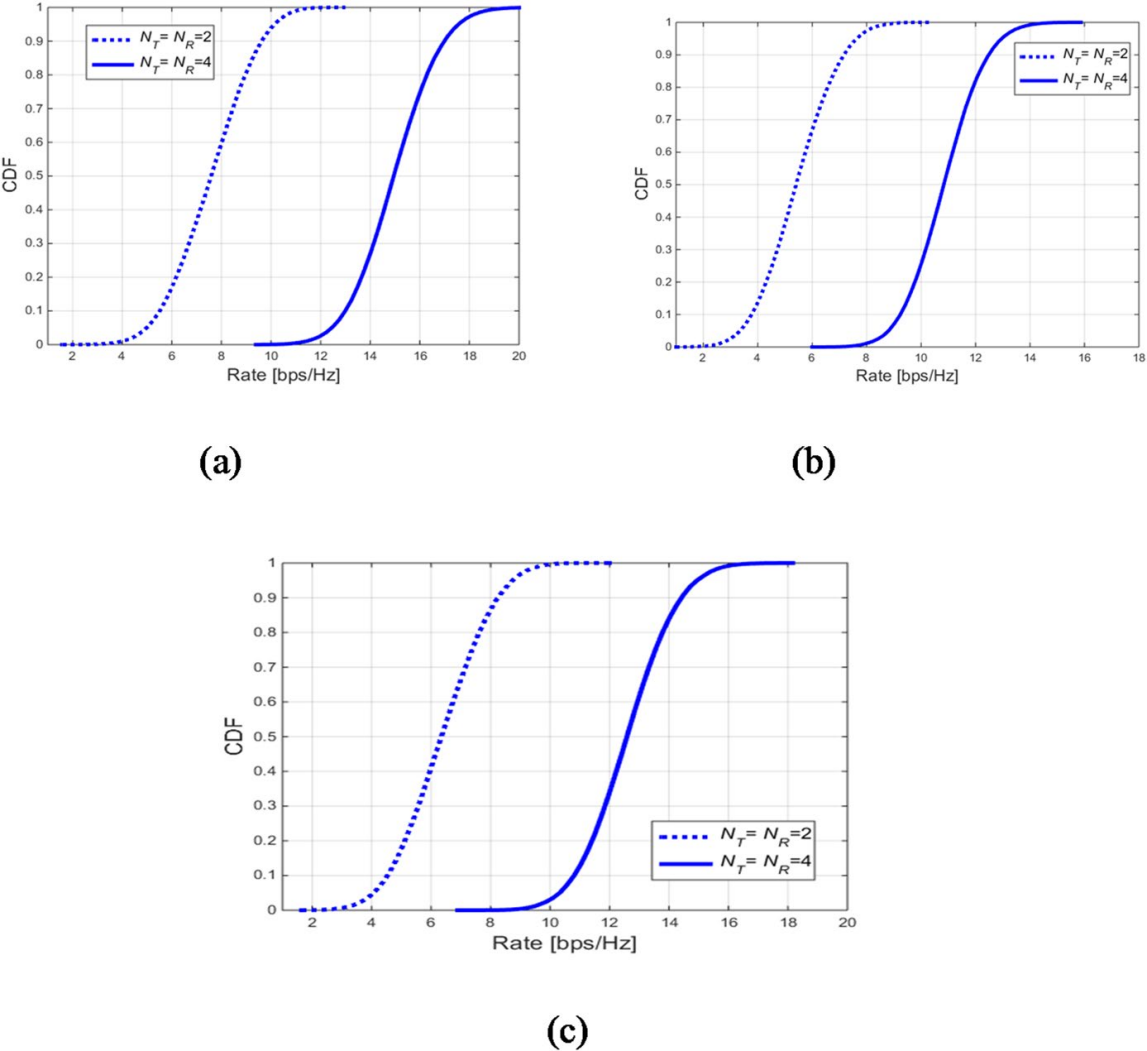
(a, b, c) where (a) CDF in opposition to bitrate without coding technique at 2 × 2, and 4 × 4 channel, **(b)** CDF against bitrate with dissimilar TCM coding method. **(c)** CDF opposed to bitrate with assorted precoding schemes by using TTCM Coding method.

Figure 6 (a, b, and c) and Table 4 compares the CDF versus bitrate performance As shown in Fig. 6(a), the uncoded systems achieve the lowest bitrates, with the 4 × 4 MIMO setup performing better than 2 × 2 due to increased spatial diversity. While Fig. 6(b) demonstrates that TCM coding provides the highest bitrate performance (≈ 15–17 bps), confirming its superior error resilience and spectral efficiency. Moreover in Fig. 6(c), the TTCM-coded system with precoding achieves moderate gains (≈ 12.8–14.8 bps) compared to the uncoded case but slightly underperforms relative to TCM because of complexity trade-offs. Overall, TCM delivers the best CDF–bitrate performance, while TTCM with precoding offers balanced but less efficient results.

**Table 4:** CDF vs. Bitrate Performance (Fig. 6a, b, c).

Fig.	Coding scheme	Channel Condition	Bitrate at CDF = 0.5 (bps)	Bitrate at CDF = 0.9 (bps)	Key observation
6(a)	No Coding	2 × 2 MIMO	~ 5.2	7	Lowest performance: Limited spatial diversity and no error correction yield minimal bitrates
6(a)	No Coding	4 × 4 MIMO	11	12.8	Improved spatial gains: 4 × 4 MIMO doubles bitrates over 2 × 2 due to increased antennas
6(b)	TCM Coding	Not specified	15	17	Optimal performance: Highest bitrates demonstrate TCM’s superior error resilience and spectral efficiency
6(c)	PAC (TTCM) Coding	Precoding + TTCM	~ 12.8	~ 14.8	Moderate gains: Precoding+ TTCM improves over no coding but underperforms vs. TCM due to complexity trade-offs

**Fig. 7:**
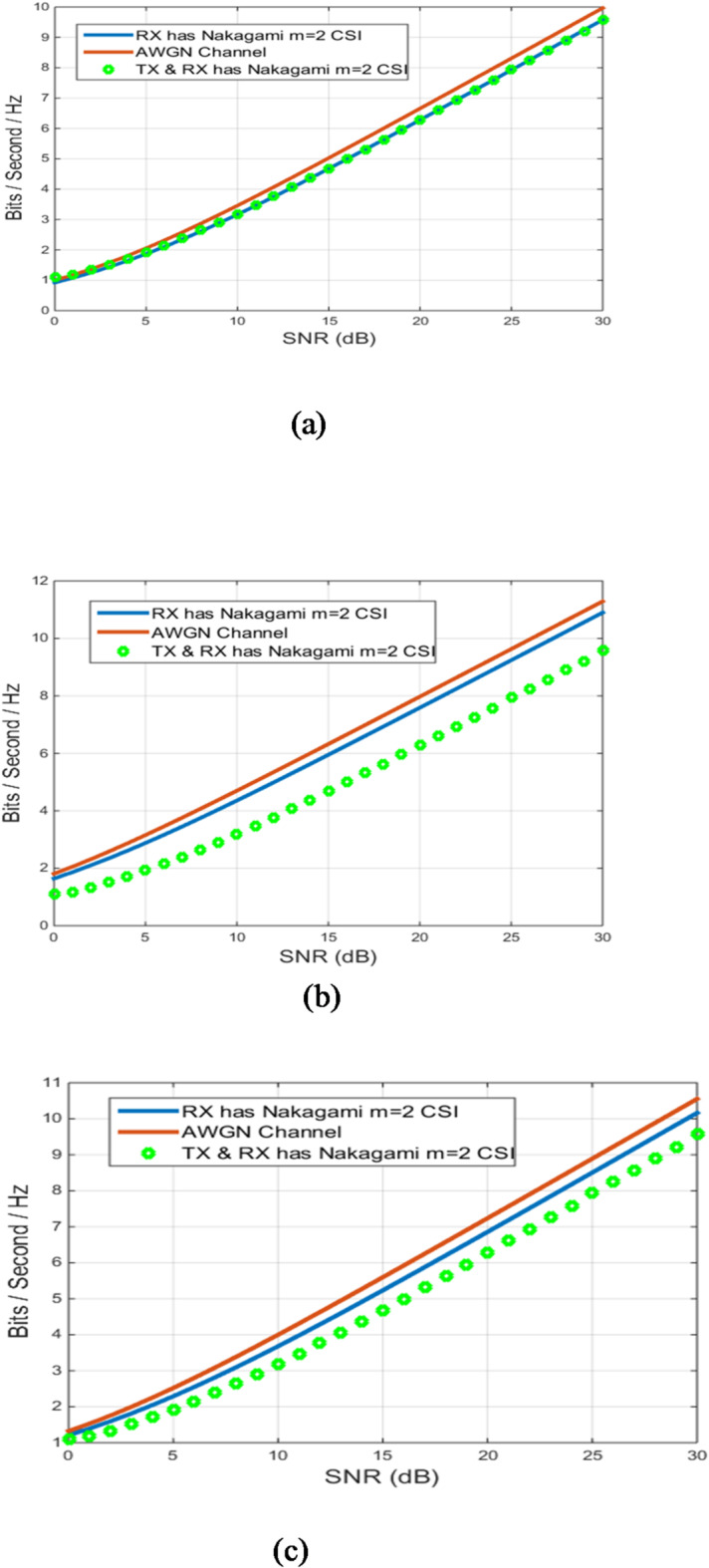
**(a**,** b**,** c) (a)** Spectral efficiency opposed to SNR without coding technique at Nakagami, and AWGN channels, **(b)** Spectral efficiency against SNR with varied TCM coding method at Nakagami, and AWGN channels. **(c)** Spectral efficiency in opposition to SNR with various precoding schemes by using TTCM Coding method at Nakagami, and AWGN channels.

Figure 7(a, b, and c) and Table 5 illustrates the spectral efficiency againt SNR performance for AWGN and Nakagami channels. Figure 7(a) shows that without coding, Nakagami channels experience a 9% reduction in efficiency compared to AWGN at 20 dB, although they show better scalability at higher SNR. Figure 7(b) shows that using TCM coding increases efficiency by about 13–15% and improves fading resistance, with Nakagami channels showing greater relative improvement. Figure 7(c) presents the TTCM with precoding configuration, which achieves balanced performance reducing the fading gap to around 7% and matching TCM’s spectral efficiency (~ 9 bps/Hz) at 25 dB. Overall, these results confirm that the use of coding and precoding techniques markedly enhances spectral efficiency and robustness across both channel environments.

**Table 5:** For Fig. 7 (a, b, c) Spectral Efficiency vs. SNR (Nakagami and AWGN Channels).

Fig.	Coding scheme	Channel type	SE@ 20 dB (bps/Hz)	SE@ 25 dB (bps/Hz)	% Improvement	Key observation
7(a)	No Coding	AWGN	~ 6.8	8.3	22.1%	Baseline with fading penalty: Nakagami shows 9% lower SE than AWGN at 20 dB, but greater SNR scalability (+ 29% vs. + 22%)
		Nakagami	~ 6.2	8	29.0%	
7(b)	TCM Coding	AWGN	8	9.7	21.3%	Optimal efficiency: Maintains 13–15% SE advantage over no coding. Higher fading resilience (Nakagami improvement: +28% vs. TCM-AWGN + 21%)
		Nakagami	~ 7.2	9.2	27.8%	
7(c)	PAC (TTCM) + Precoding	AWGN	~ 7.3	9	23.3%	Balanced performance: Precoding minimizes fading gap (7% Nakagami penalty at 20 dB). Matches TCM at 25 dB AWGN (9.0 bps/Hz) with more consistent channel scaling
		Nakagami	~ 6.8	8.5	25.0%	

**Fig. 8:**
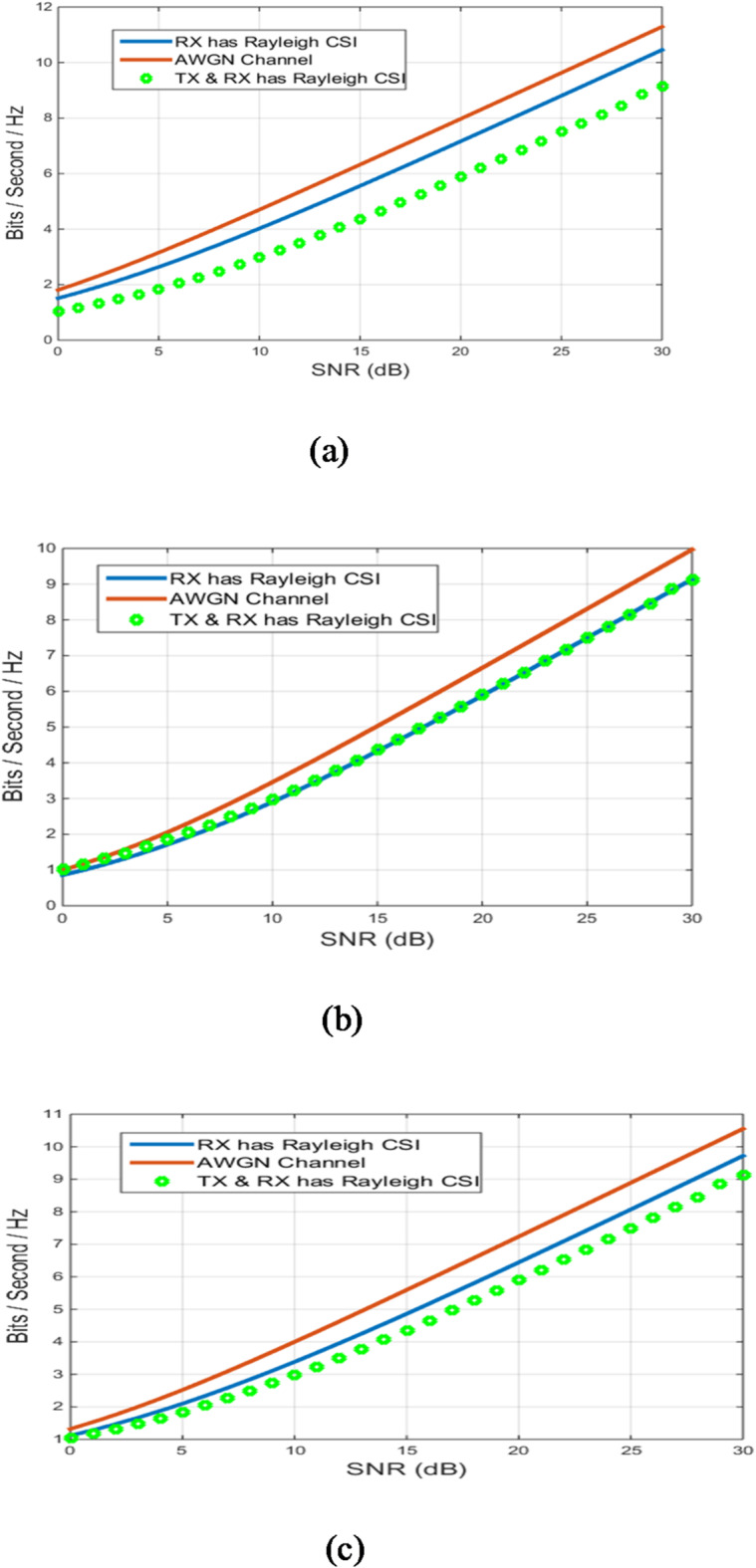
**(a**,** b**,** c) where (a)** Spectral efficiency opposed to SNR without coding technique at Rayleigh, and AWGN channels, **(b)** Spectral efficiency against SNR with assorted TCM coding method at Rayleigh, and AWGN channels. **(c)** Spectral efficiency compared to SNR with varied precoding schemes by using TTCM Coding method at Rayleigh, and AWGN channels.

Figure 8(a, b, and c) and Table 6 compares the spectral efficiency in opposition to SNR for AWGN and Rayleigh channels. Firstly, Fig. 8(a) presents the baseline case without coding, where the Rayleigh channel exhibits around a 12% performance drop compared to AWGN at 20 dB, though both show moderate improvement with increasing SNR.Then Fig. 8(b) shows that using TCM coding increases the spectral efficiency over the uncoded system by about 19%, maintaining consistently high performance and strong gain on both channels. Finally, Fig. 8(c) shows the TTCM with the precoding setup, which provides the largest absolute improvement (+ 45%) in spectral efficiency at 25 dB, but shows a lower baseline, performing about 23% less than the TCM at 20 dB. Overall, these findings confirm that while TTCM provides better scalability with precoding, TCM remains the most efficient and stable scheme in Rayleigh and AWGN environments.

**Table 6:** Spectral efficiency vs. SNR (Rayleigh and AWGN channels for Fig. 8 (a, b, c).

Fig.	Scheme	SE @ 20 dB (bps/Hz)	SE @ 25 dB (bps/Hz)	Abs. improvement (bps/Hz)	% Improvement	Key observation
8(a)	No Coding (AWGN)	~ 6.7	~ 8.4	1.7	+ 25%	Baseline performance: Rayleigh shows 12% degradation at 20 dB vs. AWGN. Moderate gains with SNR increase
	No Coding (Rayleigh)	~ 5.9	~ 7.5	1.6	+ 27%	
8(b)	TCM Coding (AWGN)	8.0	~ 9.9	1.9	+ 24%	Consistent high performance: Outperforms no coding by 19% at 20 dB. Uniform improvement across channels
	TCM Coding (Rayleigh)	~ 7.1	9.0	1.9	+ 27%	
8(c)	TTCM + Precoding (AWGN)	6.2	9.0	2.8	+ 45%	Strongest scaling but lower baseline: Best absolute gain (+ 45%), yet underperforms TCM by 23% at 20 dB
	TTCM + Precoding (Rayleigh)	6.4	8.0	1.6	+ 25%	

## Results and discussion (smart grid perspective)

TTCM is included in this study not because it consistently surpasses conventional TCM in spectral efficiency, but to illustrate the reliability–complexity trade-off inherent in iterative decoding schemes. While TCM achieves higher spectral efficiency under many scenarios, TTCM offers improved bit error rate (BER) performance, particularly in low-to-moderate Eb/N0 regimes, thanks to its turbo-based iterative decoding. This enhanced reliability is crucial for smart grid communication links supporting control, monitoring, and protection functions, where error resilience and communication integrity often take precedence over maximum throughput.

## Conclusions

In this paper, Trellis-Coded Modulation (TCM) consistently outperforms uncoded systems and Turbo Trellis-Coded Modulation (TTCM) with precoding in terms of spectral efficiency. As shown in Fig. 4; Table 2, TCM achieves approximately 27 bps/Hz, surpassing TTCM (~ 24.8 bps/Hz). In 4 × 4 MIMO channels, TCM also outperforms TTCM with precoding, achieving 15–28 bps/Hz compared to 13–24 bps/Hz for TTCM+precoding, as corroborated by CDF-bitrate analysis (Fig. 6; Table 4). At CDF = 0.9, TCM attains the highest bitrate of 17 bps.

Moreover, the results demonstrate that TCM exhibits strong robustness under fading environments, including Rayleigh and Nakagami channels. It consistently achieves a 13–19% improvement in spectral efficiency relative to the baseline reference and shows better scalability at moderate-to-high SNR levels. Comparative analysis further indicates that TCM outperforms TTCM combined with precoding in Rayleigh fading scenarios, particularly as SNR increases.

Although TTCM with precoding enhances resilience against channel impairments and provides reliability improvements compared with standalone TTCM, its achievable gains are constrained by iterative decoding complexity and associated processing overhead. These observations reveal an important practical trade-off among spectral efficiency, computational complexity, and robustness, which is a critical design consideration for Smart Grid communication infrastructures.

## Data Availability

The core of the empirical work consists of the simulation and analysis of data generated using MATLAB Code, as well as TCM and TTCM Studio simulations. This comprehensive dataset includes the source codes, numerical results, and output figures from computational models developed specifically to address the non-convex optimization problem which is formulated to enhance the achievable rate of the secondary user (SU) without exceeding both the transmitting power constraint of secondary base station and the interference temperature limit (IT) on the existing primary users (PU) in the proposed smart grid network.For any data requests related to this study, please contact Rna Ghallab (ghallabrna@gmail.com).
